# *Libidibia ferrea* (jucá) anti-inflammatory action: A systematic review of *in vivo* and *in vitro* studies

**DOI:** 10.1371/journal.pone.0259545

**Published:** 2021-11-05

**Authors:** Nayanne C. O. S. Almeida, Felipe R. P. Silva, Ana Lúcia B. Carneiro, Emerson S. Lima, José Fernando M. Barcellos, Silvania C. Furtado

**Affiliations:** 1 Graduate Program in Basic and Applied Immunology, Federal University of Amazonas, Manaus, Amazonas, Brazil; 2 Post-Doctoral Fellowship in the Graduate Program of Basic and Applied Immunology, Federal University of Amazonas, Manaus, Amazonas, Brazil; 3 Federal University of Paraíba, Paraíba, Brazil; 4 Faculty Member of Pharmaceutical Sciences, Federal University of Amazonas, Manaus, Amazonas, Brazil; 5 Department of Morphology, Institute of Biological Sciences, Federal University of Amazonas, Manaus, Amazonas, Brazil; Federal University of Santa Catarina, BRAZIL

## Abstract

*Libidibia ferrea* (Mart. ex Tul.) L. P. Queiroz (jucá) is a plant extensively used in the Brazilian folk medicine for the treatment of the inflammatory process. Primary studies have focused on the verification of these biological activities, highlighting the role of this plant in inflammatory conditions. This systematic review aimed to critically establish which part of the plant and what type of plant extract present the highest evidence of anti-inflammatory activity as *in vivo* and *in vitro* experimental models. This study has followed the recommendations by PRISMA and was registered in the PROSPERO database under number CRD42020159934. The literature review was carried out in several medical and scientific databases (Google Scholar, LILACS, ProQuest, PubMed, ScienceDirect, Scopus and Web of Science) in studies published up to February 2020 and updated on March 2021. No language restriction was made to this search. Eligibility criteria were adopted instead. The risk of bias was evaluated through SYRCLE’s RoB tool for the *in vivo* studies. 609 studies were initiated to identify the whole and the subsequent steps of screening. 13 studies remained in the results (10 *in vivo* and 3 *in vitro*). In most studies the risk of bias was low or unclear. The high risk of bias was related to the risk of attrition and reporting bias. The fruit and the aqueous extract were identified as the most used in the studies carried out on the qualitative analysis and the results of the *in vivo* and *in vitro* studies were conducive to the anti-inflammatory action, a meta-analysis could not be performed due to heterogeneity between studies and the potential risk of bias to estimate the side effects. Therefore, the implementation of *in vivo* studies following the international guidelines could collaborate with analyses of the anti-inflammatory effect of jucá.

## Introduction

*Libidibia ferrea* (Mart. ex Tul.) L. P. Queiroz, popularly referred to as pau-ferro (Brazil) or jucá (Amazon region) [1], belongs to the Fabaceae family [2]. This is a native arboreal plant occurring throughout the North [3] and Northeast [2, 4, 5] of Brazil widely used as a medicinal plant.

Several published studies have demonstrated the medicinal properties assigned to *L*. *ferrea* basis related to the extensive use of this plant in folk medicine [6], cancer chemopreventive [4, 7], hepatoprotective and antioxidant [8], anti-whitening and antiwrinkle effects [9], antileishmanial activity [10], healing, gastroprotective, antioxidant and antiulcerogenic [11] as well as analgesic and anti-inflammatory properties [12].

As described above, many studies have been conducted with *L*. *ferrea* in order to verify and confirm its biological properties. Among these studies, some have been performed in *in vivo* models [9, 12] and *in vitro* models [4]. Thus, aiming at implementing future research with less waste of resources and more optimization of time, retrospective, and systematic research help in providing the methodologies employed and results obtained.

This systematic review intends to organize and analyze scientific evidence of anti-inflammatory actions by *Libidibia ferrea* or *Caesalpinia ferrea* developing *in vivo* and *in vitro* studies. This systematic review was carried out to find answers to the following questions: Which part of the *L*. *ferrea* plant and what type of extract have the highest evidence of anti-inflammatory effects on acute inflammation using *in vivo* and *in vitro* experimental models?

Which part of the *L*. *ferrea* plant and what type of extract have the most evident anti-inflammatory effects *in vivo* and *in vitro* experimental models of acute inflammation?

## Methods

This Systematic Review followed the recommendations by Preferred Reporting Items for Systematic Review and Meta-analysis (PRISMA) [S1 and S2 Tables] and was registered in the Prospective Registry of Systematic Reviews (PROSPERO) database under protocol number CRD42020159934 (https://www.crd.york.ac.uk/PROSPERO/display_record.php?RecordID=159934).

### Search strategy

A search strategy was first performed on February 3, 2020, and updated on March 12, 2021 in the following databases: Google, Scholar, ProQuest, LILACS, PubMed, ScienceDirect, Scopus and Web of Science. The manual research was carried out in the articles included identifying a possible article that was not screened in the electronic search.

The descriptor used was divided into two groups 1. *Libidibia ferrea* OR *Caesalpinia ferrea* (intervention group) and 2. anti-inflammatory effect OR anti-inflammatory action OR anti-inflammatory properties OR anti-inflammatory. Boolean operators (AND and OR) were used to make the combinations (Search strategy) [S1 Appendix]. There was no language restriction in the systematic search from which all the references of the included studies were screened for identifying potential additional study. References were organized in Microsoft Excel^TM^ and the duplicates were removed in the same program.

### Study selection

Screening based on the information in titles and abstracts were performed by two independents blind authors classified in “yes”, “no” or “maybe”. Titles and abstracts were first read, and then, the full article. Both steps were screened applying the eligibility criteria.

Two authors (NCOSA, SCF), independently, selected the studies and collected the data. Studies showing discrepancies were settled in discussions with two other authors (ALBC, ESL).

### Eligibility criteria

PICOS criteria were established as 1. Population: Animals (*Rattus novergicus* or *Mus musculus*) or *in vitro* test; 2. Intervention: Treatment with extracts from different parts of the plant in *in vivo* and/or *in vitro* models; 3. Control: negative (saline or PBS) and positive (standard drug) controls; 4. Outcome: anti-inflammatory action; 5. Study type: experimental studies.

The inclusion criteria were published articles with non-restricted time or language; articles with titles and abstracts accorded to the research questions; *In vivo* and *in vitro* studies, which tested the anti-inflammatory action of *L*. *ferrea* or *Caesalpinia ferrea*, regardless of the tested part of the plant and the extract type. In studies, which analyzed other effects, in addition to the anti-inflammatory activity, only such data were extracted: studies that described mean and standard derivation in tables, graphs, or embedded in the texts.

The exclusion criteria for title-abstract screening were:

1. Literature reviews, systematic reviews or studies, which have not complied with the standards of Ethics Committee;
2. Studies in human beings, genetic evaluation studies or cancer model studies;
3. Phytochemical studies; morphological and anatomical studies; cytogenetic analysis; ethnobotanical studies;
4. Studies performed *in silico* or *ex vivo* models;
5. Treatment with any plant except from the *L*. *ferrea* (*C*. *ferrea*);
6. Studies based on interventions with the plant *L*. *ferrea* in non-inflammatory processes;
7. Animals with previous systematic disease, auto-immune conditions, or any other conditions, which might interfere in the inflammatory model disease evaluated such as obesity, diabetes, or pregnancy;
8. Studies without control group;
9. Toxicity, cell viability outcomes, histological data;
10. Studies without a separated control group or with unavailable data mentioned in the studies.

Besides, book chapters; encyclopedias; literature reviews; systematic reviews; conference abstracts; short communications were excluded.

Regarding the criteria related to the animal population, studies, which used mice or rats of both sexes were included. With respect to the acute inflammation model those related to paw and/or ear edema, peritonitis, vascular permeability, formally-induced paw licking, zymosan-induced arthritis, excisional wound, and wound dressing were included.

### Data collection process

Data were collected, using customized data extraction in Microsoft Excel^TM^ with the following data: First author; Year of publication; Publishing journal; Country of origin/ collection location/ or period of the year; Plant part; Extract type; Extract dose and route of administration; Type of inflammation model or type of assay; *In vivo* or *in vitro* model; Number of animals for group and cell type; Therapeutic scheme; Control used; Evaluated parameters; Results.

The variables analyzed for the two models (*in vivo* and *in vitro*) were plant collection location; plant part; extract type; inflammatory cytokines levels (TNF-α, IL-1); nitrate. Data such as mean, standard deviation and percentage were also collected.

The variables analyzed for *in vivo* model were: extract dose; route of administration; animal model (rat or mice); the number of animals for group and number of groups; paw edema volume; area under the curve (paw edema); edema ear weight; polymorphonuclear leukocyte count (PMNL); myeloperoxidase levels (MPO); malondialdehyde levels (MDA); glutathione levels; Release of vasoactive amines; peripherical inflammatory pain; plasm leakage; mast cells counting; prostaglandin E_2_ (PGE_2_); wound diameter / ulcerated area.

The variables analyzed for *in vitro* model were extract concentration; type of cell; cell assay type, control group, treatment.

### Risk of bias in individual studies

Risk of bias was conducted and evaluated by two reviewers (NCOSA, SCF). The Systematic Review Center for Laboratory animal Experimentation (SYRCLE) containing 10 entries related to six types of bias to analyze the methodological quality was used. These entries were selection bias (sequence generation, baseline characteristics, and allocation concealment); performance bias (random housing and blinding); detection bias (random outcome assessment and blinding); attrition bias (incomplete outcome data), reporting bias (selective outcome reporting) and other biases [13]. Bias information was organized in an Excel spreadsheet with the related judgments: “yes” indicates a low risk of bias, “no” indicates a high risk of bias and “unclear” indicates not sufficient information reported.

### Synthesis methods

Studies, which attended the eligibility criteria were included for narrative synthesis, thus a summarization of the collected data and descriptive analysis of the results. The data synthesis is presented at the results session. Some authors were contacted to supply some unclear or missing data.

In addition to the use of SYRCLE as described above, indirectness domain was also used to analyze the quality of evidence, following the GRADE for *in vivo* studies [14]. In addition, Grades of Recommendation, Assessment, Development and Evaluation Working Group Guideline Development Tool (GRADEpro GDT) [15] was used.

Extraction and summarized data from *in vitro* studies were described in Tables 4 and 5. To the best of our knowledge, no checklist to analyze the risk of bias validated to *in vitro* studies exists [16, 17]. Thus, there is an evaluation tool to assess the *in vitro* toxicity studies using the Science in Risk Assessment and Policy (SCIRAP tool) [18].

## Results

### Study selection

**Fig 1:**
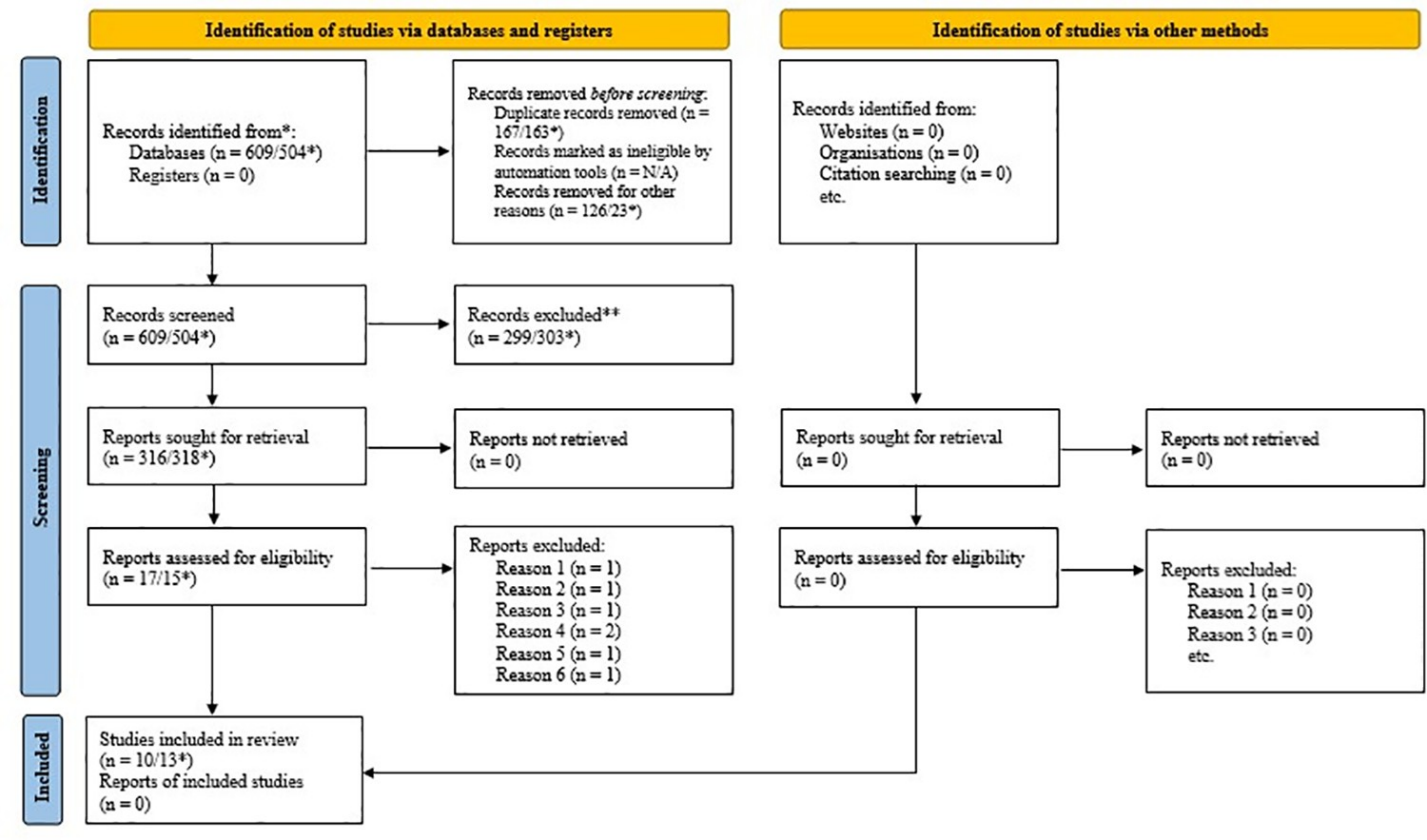
Flow diagram describing the study selections from literature searching. *Update values.

Exactly 609 studies were screened in the initial electronic search, and, after a previous screening 126 reports were excluded: encyclopedia (n = 2), book chapter (n = 16), mini reviews (n = 3), short communications (n = 8), conference abstract (n = 6), correspondence (n = 1), review article (n = 58), review (3), meeting abstract (2), review show preview for (n = 6), book chapter show preview for (n = 1), conference paper (n = 1), other (n = 19) were excluded. After this, 483 studies were considered eligible to follow up on the systematic review. From those 338 studies were from the database and 145 from grey literature. Duplicates were also removed and, after reading titles and abstracts, 17 studies were considered for full-text screening. Ten studies were considered eligible according to the eligibility criteria after the consensus by the reviewers (Fig 1).

However, this Systematic Review was actualized using the criteria described above. With this update, the research recovered 504 articles, of which 23 were excluded, there remaining 481 studies. After the removal of duplicates, 318 followed the first stage (title and abstract screening). Then, 15 remain for full-text screening. Of these 15, 10 have already been identified in the first search (February 2020). And were identified and included three more different studies (one *in vivo* and two *in vitro*) were identified and included. Two studies were excluded, in a total of 13 studies for the quantitative analysis (Fig 1).

At the first search, seven articles were included in the second phase (full article screening) and seven studies were excluded because: one presents the same genus, but it was a different species (*Caesalpinia sapan*) (reason 1), another study was a thesis which the article had already been included for data extraction and analysis (reason 2). Another study referred to a chemical characterization of *L*. *ferrea* (reason 3). Two reports were an ethnobotanical study (reason 4), one study presents the hypoglycemic activity of the plant (reason 5), and one study used the powder for the anti-inflammatory tests and not the extract (reason 6). The last two articles were the same that appeared at the update carried out on March, 2021 and they were also excluded (Fig 1).

### Study characteristics

The year of publication of the 13 articles ranged from 1996 to 2020 (Table 1). And in all studies, Brazil was the country where the plant was collected. Eleven of the studies were written in English and two in Portuguese.

**Table 1:** Plant part and type of extract from *L*. *ferrea* overview used *in vivo* and *in vitro* studies.

Plant part	Type of extract	Reference	Study type
Bark	Aqueous	[19]	*In vivo*
	Acetone-water		
Stem bark	Polysaccharide-rich (TPL-Cf)	[20]	
	Rich-polysaccharide	[21]	
Leaves	Crude aqueous	[22]	
Pods (peels and seeds)	Ethanol	[23]	
Pods (devoid of seeds)	TPL, FI, FII e FIII	[24]	
Fruits (var. *ferrea*)	Aqueous Crude	[25]	
	CE20, CE40, CE60 e CE80		
	Ethyl acetate fraction (EAF)		
	Aqueous fraction (AqF)		
Pods (var. *parvifolia*)	Crude aqueous	[26]	
	F80		
Fruits	Crude aqueous	[12]	
Mature seeds	Lipidic portion of *Libidibia ferrea*	[27]	
Fruits	Supercritical fluid extraction	[28]	*In vitro*
Stem bark	Hydroalcoholic	[29]	
Leaves	Dry (ELFLF)	[30]	

TPL-Cf (Total polysaccharides of *C*. *ferrea* barks); TPL (Total polysaccharides); FI to FIII (major polysaccharide fractions); CE 20.0–80.0% (Hydroalcoholic fractions of 20.0–80.0% ethanol); F80 (partially purified fraction); ELFLF (Lyophilizes extract from *L*. *ferrea* leaves).

Concerning studied part of the plant it was noted that: six authors used fruits [12, 23–26, 28], one used the bark [19], three used the stem bark [20, 21, 29], two used leaves [22, 30] and one used seeds [27] (Table 1).

Therefore, as regards the type of extract: the aqueous extract was performed in five studies [12, 19, 22, 25, 26], one used ethanolic extract [23], another acetonic extract [19] two rich-polysaccharides extracts [20, 21], one used four different fractions from hydroalcoholic extract CE20, CE40, CE60 e CE80% [25], one used hydroalcoholic extract [29], and one used dry extract [30]. Polysaccharides fractions [24], lipid portion of acetone extract [27], fraction 80 (F80) [26], ethyl acetate and aqueous fraction [25] and supercritical fluid [28] (Table 1).

It was observed that of the 10 *in vivo* studies included, the animals used in the experiments were Swiss mice (n = 6) [19, 21, 23, 25–27] and Wistar rats (n = 4) [12, 20, 22, 24]. Regarding the inflammation model used in the studies, there was a variety of these, and three studies performed more than one inflammation model [21, 23, 24] to verify the anti-inflammatory action of *L*. *ferrea* (Table 2).

**Table 2:** Data from *in vivo* studies included.

Author / Year	Country of Origin / Collection Country / Year Period	Route	Dose	Control group	Animal	Sex	Weight (g)	Age (days)	n. / group	Group
**Carvalho et al., 1996** [12]	Brazil / Icoaracy- Belém (PA) / Mar-1988	Oral	300 mg/kg	Indomethacin	Wistar	Both	140–170	?	13	3
**Freitas et al., 2012** [26]	Brazil / Ibimirim (PE) / Aug-2006	Oral	100 mg/kg	Saline, dexamethasone, indomethacin, piroxicam	Swiss	Both	approx. 25	approx. 50	6	6
**Lima et al., 2012** [23]	Brazil / Barbalha (CE) / Jun-2007	Oral	12.5, 25, 50 mg/kg	Saline, indomethacin	Swiss	Male	25 ± 5	50	7	5, 3, 3
**Pereira et al., 2012** [24]	Brazil / District of Custódio-Quixadá (CE)	Intravenous	0.01, 0.1, 1 mg/kg	Saline, indomethacin	Wistar	?	150–200	?	6	?
				Saline						
				Saline, methisergide, indomethacin, L-NAME						
**De Araújo et al., 2014** [19]	Brazil / PE	Oral	50, 100, 200 mg/kg	DMSO, indomethacin	Swiss	Female	25–35	?	6	11
**Sawada et al., 2014** [27]	Brazil / Joanes, Salvaterra, Marajó Island (PA) / 2011	Oral	10 mg/kg	Saline, indomethacin	Swiss	Male	30–35	56	10	5
**Pereira et al., 2016** [20]	Brazil / Custódio-Quixadá District (CE) / May-2013 and Feb-2014	Topic	0.025–0.1%	Saline, collagenase ointment	Wistar	Male	180–200	61	16	6
**Falcão et al., 2019** [22]	Brazil / Caatinga Biome in Recife (PE) / Sept-2014	Oral	100, 200, 300 mg/kg	Normal (without zymosan treated with 50 mg saline 0.9%) / zymosan + salina) / diclofenac (100 mg/kg)	Wistar	Male	150 ± 250	?	6	6
**Falcão et al., 2019** [25]	Brazil / Limoeiro (PE)	Oral	50, 100, 200 mg/kg	Saline, diclofenac	Swiss	Male	40 ± 2.0	60	6	9
**Holanda et al., 2020** [21]	Brazil / District of Custódio (Quixadá/CE)	Intravenous	0.001, 0.01, 0.1 and 1 mg/kg	NaCl and zymosan	Swiss	Female	25–35	?	8	?
		Oral	1 mg/kg	Ascorbic acid, zymosan						

?: data not found; mg/kg: milligram/kilo; g: gram; NaCl: sodium chloride; approx.: approximately.

The most used route of administration for treatment was the orally (n = 8) [12, 19, 21–23, 25–27]. Other types of routes of administration present in the other studies were intravenous (n = 2) [21, 24], topical [20]. In all preclinical experimental models, anti-inflammatory activity was suggested independently of the plant and it was independent of the animal model, part of the plant and the type of extraction or fraction used in the studies. This potential action was observed through a reduction/inhibition of paw edema volume [12, 21, 24], reducing/migration from the number of PMNL [19, 21–26], reduction of ear edema [23], inhibition of vascular permeability [20, 23], reduction in the number of licks [27], reduction of wound area [20], evaluation of inflammatory mediators [20–22, 25] (Table 3).

**Table 3:** Data from outcomes of *in vivo* studies included.

Author / Year	Anti-inflammatory model	Measure parameter	Anti-inflammatory activity results	Measurement (Mean ± S.E.M. / S.D.)		Measurement (%)	
**Carvalho et al., 1996** [12]	Carrageenan-induced paw edema	Paw volume (mL)	Paw edema reduction in the 2^nd^ and 3^rd^ hours	?		Extract: 36.3% (2^nd^ hour) / 23.0% (3^rd^ hour)	Indomethacin: 61.0% (2^nd^ hour) / 64.6% (3^rd^ hour)
**Freitas et al., 2012** [26]	Carrageenan-induced peritonitis	PMNLs counting (polymorphonuclears leucocytes) (x10^6^/mL)	Exudate inflammatory reduction in number of PMNL	(PMNL/mL exudate ± S.E.M) CE: 5.8 ± 0.3 x 10^6^ / F80: 38.6.0 ± 0.1 x 10^6^		CE: 40.9% /F80: 38.2%.	Indomethacin: 72.2%; Piroxicam: 46.7%; Dexamethasone: 68.1%.
**Lima et al., 2012** [23]	Thioglycolate induced peritonitis	Total number of leukocytes (x10^6^)	Inhibition inflammatory response	(S.D.) 25 mg/kg: 4.14 ± 0.5 x 10^6^ / 50 mg/kg: 3.69 ± 0.5 x 10^6^		25 mg/kg: 68.4% / 50 mg/kg: 71.8%	
	Xylene-induced ear edema	Ear weight (mg)	Ear edema reduction	(S.D.) 50 mg/kg: 5.5 + 1.2 mg		50 mg/kg: 66.6%	Indomethacin: 83.9%
	Vascular permeability induced by acetic acid	Release of vasoactive amines and formation of edema (nm)	Inhibition vascular permeability	?		50 mg/kg: 66.1%	
**Pereira et al., 2012** [24]	Carrageenan-induced paw edema	Paw volume difference of displacement (mL) and area under curves—AUC (arbitrary units %); Plasma leakage (mg Evans’s blue/g).	Inhibition of paw edema, antiedematogenic activity	(S.E.M.) TPL 1 mg/kg: 60–180 min (23 ± 2.86 AUC) / 180–300 min (6 ± 2.14 AUC) / FIII 1 mg/kg: 60 min—0.28 ± 0.06 mL		TPL 1mg/kg: 60.0% (max. inhibition) / 48.0% (initial) / 76% (late) / FIII: 60 min—53.0% 300 min—85.0%	Indomethacin: 64.0% (initial) / 53.0% (late)
	Paw edema induced by dextran			(S.E.M.) FIII 1 mg/kg: 30 min—38.45 ± 8.66 AUC / 180 min: 0.08 ± 0.03 mL		FIII 1 mg/kg: 30 min– 53.0%, 180 min—70.0%	
	Paw edema induced by PGE2, L-arginine			?		PGE2—FIII: 63.0% L-arginine—FIII: 61.0%	
	Paw edema induced by Histamine, Serotonin, Bradykinin, 48/80 compound			?		Bradykinin—FIII: 60.0%. Histamine—FIII: 65.0%; 48/80 Compound—FIII: 36.0%; Serotonin—FIII: 62.0%	
	Carrageenan-induced peritonitis	Count of total and differential leukocytes (neutrophils, eosinophils, mast cells, mononuclear) (×10^3^/mL) and proteins (mg/mL) in peritoneal fluid.	Inhibition of leucocyte counting	(S.E.M.) FIII 1 mg/kg: 2.24 ± 0.03 x 10^3^ carrageenan: 6.23 ± 0.07 x 10^3^		FIII: 64.0%	
	Peritonitis induced by fMLP			FIII 1 mg/kg: 0.54 ± 0.04 x 10^3^ fMLP: 4.39 + 0.49 x 10^3^		FIII: 88.0% fMLP: 84.0%	
	Anti-inflammatory activity evaluation	Mast count	Degranulated mast cells (late phase)	240 min—FIII: 0,56 ± 0,05 mL / 300 min—FIII: 0,43 ± 0,05 mL		L-NAME: 84.0% Methisergide: 84.0%	
**De Araújo et al., 2014** [19]	Carrageenan-induced peritonitis	Total leukocyte count (total number of cells per peritoneal cavity)	Leukocyte migration reduction	?		?	
**Sawada et al., 2014** [27]	Formalin-induce licking (inflammatory pain)	Number of licks induced with formalin/ Evaluation of the mechanism of action LPLF seeds	Licks quantitative reduction	?		74 ± 2% in early phase, 100% late phase—maximal inhibition	Indomethacin: 76 ± 7% in late phase
**Pereira et al., 2016** [20]	Wound model	Wound area (mm^2^), (wound closure %), Vascular permeability vascular (nm), Inflammatory mediators (IL-1β, PGE2, TNF-α; MPO, Nitrate, MDA)	Wound area reduction, leukocyte infiltration and vascular permeability	(S.E.M.) TPL-Cf 0.1%: 38.99 ±1.9 mm^2^ in area reduction and increase on the wound at the 2^nd^ day. TPL-Cf—IL-1: reduction of 2.20 ± 0.03 pg/mL, at 2^nd^ day/ PGE_2_: 0.0062 ± 0.001 reduction at 7^th^ day. / Nitrite: 26.86 ± 9.5 μM increase at 5^th^ day / MPO: 41.28 ± 4.66 U/mg tissue (2^nd^ day) and 19.67 ± 8.18 U/mg tissue (5^th^ day) reduction / MDA: 937.6 ± 72.36 μM/g tissue reduction at 5^th^ day / Vascular permeability: 29.08 ± 4.18 (2^nd^ day) and 26.44 ± 4.18 mg Evans Blue/g tissue (5^th^ day) reduction. Collagenase - 2^nd^ day 38.27 ± 1.3 / 5^th^ day 29.22 ± 1.9 / 7th 7.08 ± 0.8 mm^2^		TPL-Cf: 29.0% (2nd day) and 26.0% (5th day) reduction of polymorphonuclear infiltration / IL-1: reduction 42.0% at 2^nd^ day / PGE_2_: reduction 73.0% at 7^th^ day / MPO: 53.0% (2^nd^ day) and 63.0% (5^th^ day) reduction / MDA: 38.0% / Vascular permeability reduction: 48.0% (2^nd^ day) and 52.0% (5^th^ day)	
**Falcão et al., 2019** [22]	Zymosan-induced arthritis	Cellular influx (global leukocyte counting (mm^3^), MPO (U/mL), MDA (nmol/mL), Glutathione (nmol/mL), Inflammatory cytokines [IL-1β (pg/mL) / TNF-α (pg/mL)]	Leukocyte influx reduction from synovial fluid, reduction of the levels from IL-1β, TNF-α, MPO, MDA, glutathione increase	?		(S.E.M.) Extract reduced leukocyte influx in 76 ± 2% at the 100, 200 and 300 mg/kg doses / MPO: reduction in approx. 85% + 7% / Glutathione levels increased: 41 nmol/mL / MDA levels reduced: 60.0% (200 e 300 mg/kg)	Diclofenac: 56%
**Falcão et al., 2019** [25]	Carrageenan-induced peritonitis	MPO (U/μL.), MDA (nmol/μL) and glutathione total levels (nmol/μL) / leukocyte numbers (x10^3^)	Leucocyte migration reduced in all preparations, Reduction in MPO and MDA levels, increase in glutathione levels	?		?	
**Holanda et al., 2020** [21]	Zymosan induced paw edema	Paw volume (mL) or area under the curve -AUC / MPO (U/mg tissue)	Paw edema inhibition, reduction in MPO levels	PE-Cf 1 mg/kg 58 ± 9 mL (4h), 52 ± 10 mL (5h) / 1-3h - PE-Cf 0.1 mg/kg: 220 ± 5 AUC, PE-Cf 1 mg/kg: 140 ± 16 AUC / 3–6 h—PE-Cf 0.01 mg/kg: 580 ± 15 AUC, PE-Cf 0.1 mg/kg: 331 ± 15 AUC, PE-Cf 1 mg/kg: 182 ± 18 AUC; MPO—PE-Cf 1 mg/kg: 17 + 1 U/mg		PE-Cf 1 mg/kg 71.0% (4h), 74.0% (5h) / 1-3h - PE-Cf 0.1 mg/kg: 39.0%, PE-Cf 1 mg/kg: 61.0% / 3–6 h—PE-Cf 0.01 mg/kg: 43.0%, PE-Cf 0.1 mg/kg: 36.0%, PE-Cf 1 mg/kg: 69.0% / MPO—PE-Cf 1 mg/kg: 43.0%	
	Peritonitis induced by zymosan (i.v.)	Leukocyte migration (total leukocyte, neutrophil, mononuclear) (mm^3^) / GSH (μmol/mL–A_412_ nm) / GPx (U/mg proteins–A_340_ nm) / Nitrate (mM–A_540_ nm) / MDA (U/mL–A_535_ nm)	Leukocytes and neutrophils reduction. Increase in GSH e GPx levels, reduction n NO_2-_/No_3-,_ MDA levels	PE-Cf 1mg/kg—Leukocytes 1.063 ± 130 mm^3^, neutrophils 432 ± 45 mm^3^ / GSH: 736 ± 65 μmol/mL / GPx: 0.037 ± 0.007 U/mg protein / NO_2-_/NO_3-_: 0.131 ± 0.033 mL e MDA: 98 ± 10 U/mL		PE-Cf 1mg/kg—Leucocytes 69.0% / neutrophils 84.0% / GSH: 65.0% / GPx: 72.0% / NO_2-_/NO_3-_: 73.0% / MDA: 37.0%	
	Peritonitis induced by zymosan (p.o.)	Leukocyte migration (mm^3^)	Inhibition of leukocyte and neutrophils migration	PE-Cf 1mg/kg–Leucocytes: 2.143 ± 123 mm^3^, neutrophils: 742 ± 75 mm^3^	Zymosan: 3.149 ± 23/mm^3^	PE-Cf 1mg/kg–Leucocytes: 41.0%, neutrophils 76.0%	

? (Data not demonstrated); S.E.M. (standard error of the mean); S.D. (standard deviation); min (minute); mL (milliliter); CE (Crude aqueous extract); F80 (partially purified fraction); TPL (Total polysaccharides); FI-FIII (major polysaccharide fractions); LPLF (Lipidic portion from *Libidibia ferrea*); TPL-Cf (Total polysaccharides of *C*. *ferrea* barks); g (gram); CE20-CE80 (Hydroalcoholic fractions of 20.0–80.0% ethanol); fMLP (N-formyl-methionyl-leucyl-phenylalanine); LfAE (Crude aqueous extract of *L*. *ferrea*); mg (milligram); kg (kilo) PGE2 (Prostaglandin E_2_); PMNL (polymorphonuclears leucocytes); h (hour); AUC (area under curve); p/v (weight/volume); i.p. (intraperitoneal); *p*.*o*. (per oral); COX-2 (cyclocoxygenase-2); nm (nanometer); ng (nanogram); μg (microgram); μL (microliter); U/μL (units/microliter); nmol/μL (nanomole/microliter); MPO (myeloperoxidase); MDA (malondialdehyde); TNF-α (Tumor necrosis factor alpha); IL-1 (Interleukin 1); mm^3^ (cubic millimeters), NaCl (sodium chloride); PE-Cf (Rich-polysaccharides extract of *Caesalpinia ferrea* stem bark); GHS (Reduced glutathione); GPx (Glutathione peroxidase); ~ (about).

In the *in vitro* studies, the predominant cell type was the RAW cells 264.7 macrophages [28, 29], Balb/3T3 clone A31 fibroblasts [28], BV2 microglial cell [30], monocytes of human peripheral blood [29] (Table 4). The identification of anti-inflammatory action was verified by identifying inflammatory mediators (Table 5).

**Table 4:** General characteristics of *in vitro* studies.

Author / Year	Country of Origin / Collection Country / Year Period	Extract concentration	Control group	Cellular type	Assay type
**DIAS et al., 2013** [28]	Portugal / Belém do Pará (Brazil)	30 mg/mL (vol 5 μL)	Negative: without LPS or sample / Positive: com LPS	RAW 264.7 macrophage and Balb/3T3 clone A31 fibroblasts (ATCC, Manassas) 1x10^5^ / 2 mL	LPS-induced inflammation
**NETO, 2018** [30]	Brazil / Pici Campus—Fortaleza (CE) / Mar, 2017	1 mg/mL (150 μL)	Control: 100 μL Griess reagent	BV2 microglial cells from rats’ brain, retrovirus transformed (1 x 10^6^ cells/mL)	Nitrite determination / LPS induced neuroinflammation
**LINS, 2020** [29]	Brazil / AM	7.5% (w/v) (1.56; 3.12; 6.25; 12.5; 25; 50; 100 μg/mL)	Negative: DMEM / Positive: LPS from *E*. *coli* 1 μg/mL / Standard drug: Dexamethasone	RAW 264.7 macrophages (10^6^ cells/mL)	Nitrite quantification / LPS from *Escherichia coli*
			Negative: RPMI 1640/ Positive: LPS de *E*. *coli* 1 μg/mL / Standard drug: Dexamethasone	Peripherical human blood monocytes (2x10^6^ cells/mL)	

mg/mL (milligram/milliliter); ATCC (*American Type Culture Collection*); LPS (lipopolysaccharides).

**Table 5:** Outcome description from the *in vitro* studies.

Autor / Year	Treatment	Parameter evaluated	Results
**DIAS et al., 2013** [28]	Cell culture in DMEM-F12 HAM medium with phenol red medium in 24-well plate and were pre-incubated with samples of each dressing (approximately 1cm^2^) without load or extract, after 20 mL of LPS was added to the medium. 2, 6, 24, and 72 h collection of an aliquot of 500 mL.	Quantification of the amount of extract loaded/released (gravitationally) / cytocompatibility / Production of IL-1α and TNF-α (ELISA) / Nitric Oxide Concentration (quantification curve 0–15 mM); LDH cytosolic enzyme released in the culture medium	LDH test: demonstrated low cell viability after 72 h / Levels of TNF-α increases progressively as a function of time from 2 to 24 hours, while IL-1α levels increase in two hours.
**NETO, 2018** [30]	Cell suspension incubated in 96 well plates for 24 h. ELFLF extract was added. After 1 h was challenge with LPS. 100 μL of Griess reactive was added.	Nitrite quantification (NO) (standard curve 15 μM a 1000 μM)	NO levels formation was significative reduced by 50 μg/mL. p < 0.05
**LINS, 2020** [29]	RAW 264.7 macrophage was sanded in DMEM medium in 96 well plates. Culture medium was removed, and the cells was challenged with 1 μg/mL– 50 μg/well of LPS. Cells was treated with *L*. *ferrea* extract (1.56, 3.125, 6.25, 12.5, 25, 50 and 100 μL/well). Cells with LPS was incubate for 24h. Three experiments were made with triplicates.	Nitrite determination (standard curve)	Compared to dexamethasone and LPS, 50 e 100 μg/mL better reduced the NO levels. p < 0.05
	Human monocytes were sanded in RPMI medium in 96 well plates. Same procedure of RAW 264.7 macrophage.		All concentration inhibition the NO levels, although 50 and 100 μg/mL were better than the other concentrations. p < 0.05

LPS (lipopolysaccharides); cm^2^ (square centimeters); mL (milliliters); mM (milimolar); LDH (lactate dehydrogenase); h (hour); TNF-α (Tumor necrosis factor alpha); IL (interleukin); ELISA (Enzymatic immunoadsorption assay); μg/mL (microgram per mL); NO (nitric oxide); ATCC (American Type Culture Collection); DMEM-F12 (Dulbecco’s Modified Eagle Medium: Nutrient mixture F-12); RPMI (Roswell Park Memorial Institute).

### Risk of bias in individual studies

The outcomes evaluate the risk of bias in *in vivo* studies. Therefore, when there was a similarity between the studies, the analysis was executed once, and when there was any different outcome, this was separably analyzed (Table 6).

**Table 6:** Risk of bias *in vivo* studies according to SYRCLE’s RoB tool of the ten studies included in the systematic review.

Study	Inflammatory model	Selection bias			Performance bias		Detection bias		Attrition bias	Reporting bias	Other
		1	2	3	4	5	6	7	8	9	10
Carvalho et al., 1996	Paw edema (carrageenan)	?	Y	?	Y	?	?	?	N	Y	Y
Freitas et al., 2012	Peritonitis (carrageenan)	?	Y	?	Y	?	?	?	Y	Y	Y
Lima et al., 2012	Peritonitis (thioglycolate)	?	Y	?	Y	?	?	?	Y	N	Y
	Ear edema (xylene)	?	Y	?	Y	?	?	?	Y	Y	Y
	Vascular permeability	?	Y	?	Y	?	?	?	Y	Y	Y
Pereira et al., 2012	Paw edema (carrageenan)	?	Y	?	Y	?	?	?	Y	Y	Y
	Paw edema (dextran)	?	Y	?	Y	?	?	?	Y	Y	Y
	Paw edema (histamine)	?	Y	?	Y	?	?	?	Y	N	Y
	Paw edema (serotonin)	?	Y	?	Y	?	?	?	Y	N	Y
	Paw edema (48/80 compound)	?	Y	?	Y	?	?	?	Y	N	Y
	Paw edema (bradykinin)	?	Y	?	Y	?	?	?	Y	N	Y
	Paw edema (PGE-2)	?	Y	?	Y	?	?	?	Y	N	Y
	Paw edema (L-arginine)	?	Y	?	Y	?	?	?	Y	N	Y
	Peritonitis (carrageenan)	?	Y	?	Y	?	?	?	Y	N	Y
	Peritonitis (fMLP)	?	Y	?	Y	?	?	?	Y	N	Y
	Inflammatory evaluated	?	Y	?	Y	?	?	?	Y	Y	Y
De Araújo et al., 2014	Peritonitis (carrageenan)	?	Y	?	Y	?	?	?	Y	N	Y
Sawada et al., 2014	Licking	Y	Y	?	Y	?	?	?	Y	Y	Y
Pereira et al., 2016	Wound	?	Y	?	Y	?	?	?	Y	Y	Y
Falcão et al., 2019	Arthritis (zymosan)	?	Y	?	Y	?	?	?	Y	Y	Y
Falcão et al., 2019	Peritonitis (carrageenan)	Y	Y	?	Y	?	?	?	Y	Y	Y
Holanda, 2019	Paw edema (zymosan)	?	Y	?	Y	?	?	?	Y	Y	Y
	Peritonitis (i.v.) (zymosan)	?	Y	?	Y	?	?	?	Y	Y	Y
	Peritonitis (p.o.) (zymosan)	?	Y	?	Y	?	?	?	Y	Y	Y

Y (YES) = low risk of bias; N (NO) = high risk of bias,? = Unclear bias. Sequence generation (1), Baseline characteristics (2), Allocation concealment (3), Random housing (4), Blinding (5), Random outcome assessment (6), Blinding (7), Incomplete outcome (8), Selective outcome reporting (9) and others (10).

Note: Scale was adapted according to the use of different *in vivo* experimental models of inflammation.

Following the SYRCLE’s RoB tool, the following risk of bias presents: eight studies with unclear selection bias risk [12, 19–24, 26] since they only described that they were divided into groups, not stating whether they have been randomized or not. The other two studies described that the animals have been randomized but have not informed the method used to take such step [25, 27]. They were, then, judged as having a low risk of bias (1).

All *in vivo* studies present a low risk of bias regarding baseline characteristics, in other words, the animals were induced to the inflammatory condition after which, they were given treatment [12, 19, 21–26] or induced to wound [20] before treatment application (2). As to allocation concealment the risk was considered unclear for all *in vivo* studies for lack of sufficient information with respect such concealment (3).

Concerning the risk of performance bias, all studies have been categorized as low risk of bias. This type of bias refers to random housing as they have been maintained in baseline conditions before the beginning of the experiment, such as the provision of water and food (4). Yet, as to blinding (5) there was no evidence as to whether the researchers who manipulated the animals had any knowledge of what group was the control or the treatment group.

Regarding detection bias, both the random evaluation of the outcome (6) and blinding (7) were described as uncertain, since it was not mentioned in the primary studies whether the analysis of the outcomes was performed randomly or whether those who analyzed the outcomes were random. In the analysis of the risk of frictional bias (8), it has been observed that no study has reported an animal loss during the experiment. Carvalho et al. (1996) described the division of two groups of animals in the methodology, however, in the results, they presented three groups, that is, they included a negative control group [12].

Nine studies reporting bias [12, 20–27] described all outcomes related to the reporting bias risk (9). However, De Araújo et al. (2014) related acetonic and aqueous extract of *L*. *ferrea* extract on the discussion without apparent description of the anti-inflammatory action of this results in isolation [19].

At the peritonitis experiment [23] the ethanolic extract dose (12,5 mg/kg) received more description than the other doses (25 e 50 mg/kg). About other sources of bias (10) all studies were classified as low risk of bias. Although, two studies have not shown the ethics committee number [12, 27].

### Certainty of evidence

The analysis of the uncertainty of inconsistency, publication bias, inaccuracy and *in vivo* studies were presented in a narrative description:

Imprecision: It was observed that there is a heterogeneity in the studies, such as the size of the samples and amounts of groups used by experiments; the metrics of variation, in most studies, was through mean ± SEM [20–22, 24–28], mean ± standard deviation [23] and the expression of volume difference [12]. In all *in vivo* studies the calculation of the sample size was not detected. Even with these inconsistencies the studies tended to present the same direction of the effect, that is, *L*. *ferrea* anti-inflammatory activity, so the certainty of the evidence would not downgrade [S2 Appendix].

Publication bias related to the included studies: only one study [23] described in the topic of funding by agencies, which have supported the work. This topic was not requested in the journal in the other studies. Therefore, many added this funding information in the acknowledgement, and none presented to be funded by any industry. In four studies [12, 24, 26, 27] the topic of conflict of interest was not required in the journal. In the other six studies [20–23, 25, 28] the topic was dealt with, and a conflict of interest was identified. With this information it can be considered that the publication bias was apparently undetected, given the existing level of uncertainty. All *in vivo* studies were published in a scientific journal [S2 Appendix].

Since the meta-analysis was not performed, the inconsistency was not required to be taken into account. Considering the conditions, which could affect the outcome, apparently all performed the housing and apply water and food regimes *ad libitum*. In all studies were identified that the animals were acclimatization, describing at least the temperature, only in one study was not detected this information [27] [S2 Appendix].

Indirectness: As to the research question it was observed that the part of the plant most frequently used in the experiments was the fruit and the mostly used extract was the aqueous extract. As all studies presented anti-inflammatory activity, it can be inferred that those are the ones that showed the greatest evidence of this action, regardless of the experimental model used. Usually, teas/infusions are administered after the appearance of some inflammatory process in humans. However, excepted one study [20] almost all the other studies have induced the inflammatory process after plant administration. Thus, the certainty of evidence should be downgraded [S2 Appendix].

Based on the GRADE criteria the certainty of evidence for *in vivo* studies was also evaluated. Only one outcome was considered high [20], others were considered with moderate certainty [21–27] and low certainty [12, 19, 24]. Further information can be found at Table 7.

**Table 7:** Certainty of evidence from *in vivo* studies.

Outcome	Certainty assessment					Certainty
	Risk of bias	Inconsistency	Indirectness	Imprecision	Other considerations	
Inflammation inhibition (paw volume) [12]	serious^a^	not serious	serious	not serious	none	ꚚꚚꓳꓳ low
Cellular migration reduction (PMNL counting) [26]	not serious	not serious	serious	not serious	none	ꚚꚚꚚꓳ moderate
Inhibition of cellular migration [23]	not serious	not serious	serious^b^	not serious	none	ꚚꚚꚚꓳ moderate
Ear edema reduction [23]	not serious	not serious	serious	not serious	none	ꚚꚚꚚꓳ moderate
Vascular permeability inhibition [23]	not serious	not serious	serious	not serious	none	ꚚꚚꚚꓳ moderate
Paw edema inhibition (carrageenan; dextran) [24]	not serious	not serious	serious	not serious	none	ꚚꚚꚚꓳ moderate
Paw edema inhibition (histamine; serotonin; bradykinin, PGE-2; L-arginine; compound 48/80) [24]	serious^b^	not serious	serious	not serious	none	ꚚꚚꓳꓳ low
Peritonitis (carrageenan; fMLP) [24]	serious^b^	not serious	serious	not serious	none	ꚚꚚꓳꓳ low
Inflammatory evaluated [24]	serious^b^	not serious	serious	not serious	none	ꚚꚚꓳꓳ low
Total leukocyte count [19]	serious^b^	not serious	serious	not serious	none	ꚚꚚꓳꓳ low
Number of licks induced with formalin [27]	not serious	not serious	serious	not serious	none	ꚚꚚꚚꓳ moderate
Wound area reduction [20]	not serious	not serious	not serious	not serious	none	ꚚꚚꚚꚚ high
Cellular migration reduction [22]	not serious	not serious	serious	not serious	none	ꚚꚚꚚꓳ moderate
Reduction of cell influx [25]	not serious	not serious	serious	not serious	none	ꚚꚚꚚꓳ moderate
Paw edema inhibition [21]	not serious	not serious	serious	not serious	none	ꚚꚚꚚꓳ moderate
Leukocytes and neutrophils reduction [21]	not serious	not serious	serious	not serious	none	ꚚꚚꚚꓳ moderate
Inhibition of leukocyte and neutrophils migration [21]	not serious	not serious	serious	not serious	none	ꚚꚚꚚꓳ moderate

^a.^ Most domains presented uncertain risk of bias; It was not detected the ethics committee number or if the animals were randomized.

^b.^ It was not detected the animal randomization. Most domains presented uncertain of bias.

*In vitro* studies: SciRAP [18] was used with adaptations as a tool for the evaluation of the quality of reports. Five aspects (test compound and controls, test system, administration of test compound and data collection and analysis) were presented, with 23 topics on the whole. Items related to the compound used chemical (item 1), purity of the compound (item 2), solubility of the test compound (item 3) (test compound and controls); system source (item 7), metabolic competition (item 8) were removed since these items are related to the toxicity of the compound (test system); effect of the compound test on cytotoxicity (item 19) since this was not the focus of the study (data collection and analysis).

**Fig 2:**
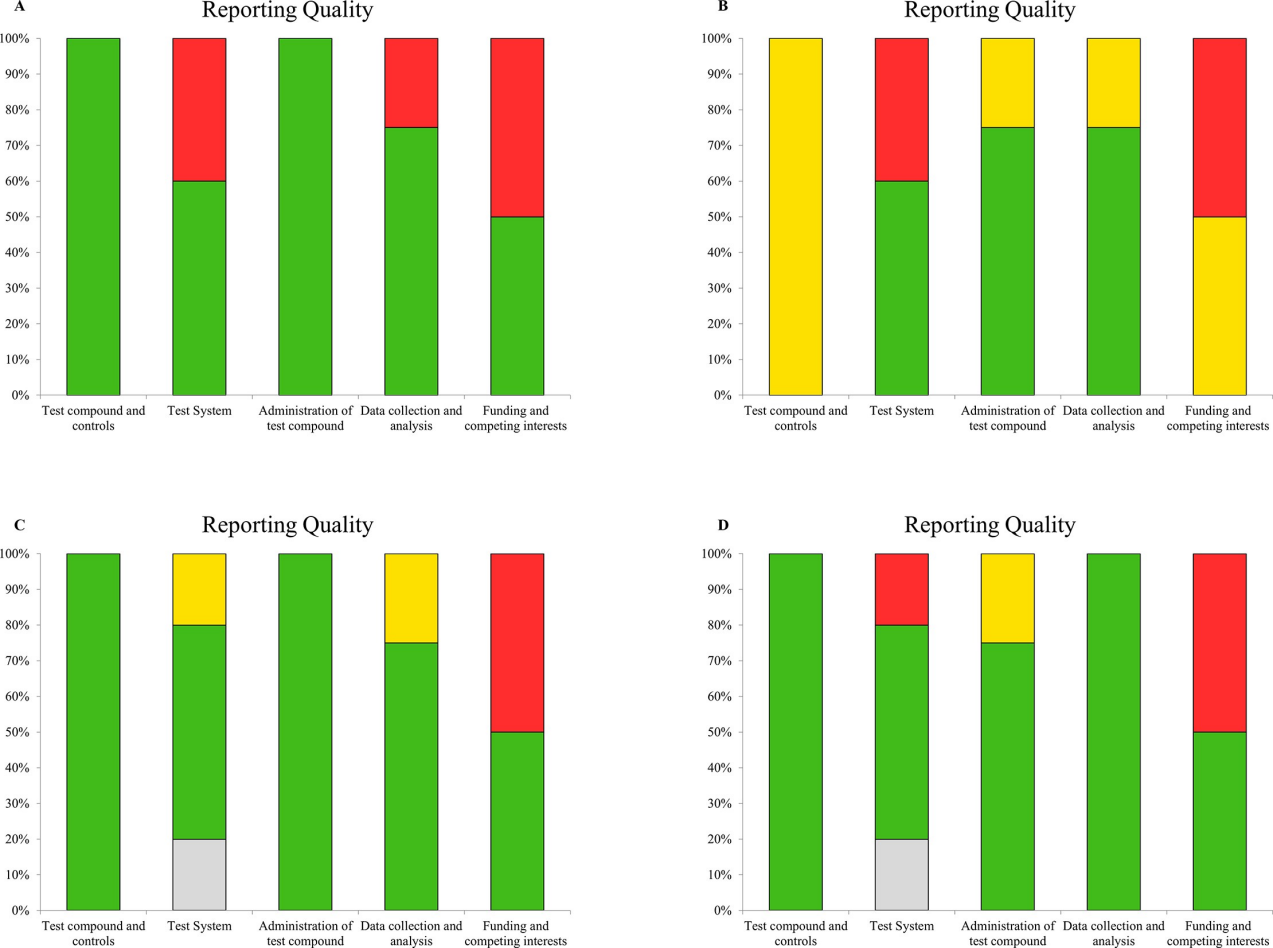
Reporting quality in *in vitro* studies. A. *L*. *ferrea* fruits quality reporting on *in vitro* study. B. *L*. *ferrea* leaves quality reporting on *in vitro* study. C and D. *L*. *ferrea s*tem bark quality reporting on *in vitro* study. Grey: not determined; green bar: fulfilled; yellow bar: partially fulfilled; red bar: not fulfilled.

With respect to test and control compound, studies have been analyzed under the items associated to the description of the vehicle, and to the untreated control or the vehicle if they were analyzed as fulfilled [28, 29] and partially fulfilled [30]. As to the item test system, the identification of the cell line/cell type in which all studies presented this information (fulfilled) were analyzed. Apparently, only one study has described the days in which cell passages to one of the cell line [29] have taken place. In the other studies no identification was possible. Information on the screening of contamination was not identified in the studies. They were presented as undetermined [28–30] and not fulfilled [29] (Fig 2A–2D).

In the item administration of test compounds concentrations or doses, cell densities and number of replicates have been described in all studies (completed). The duration of the treatment was considered as fulfilled [28, 29] and partially fulfilled [29, 30] (Fig 2A–2D).

Data collection and analysis, if the tests and/or analytic methods were sufficient to describe the results, the criterion was considered as fulfilled [28, 29], partially fulfilled [30]. Time point for the data was considered fulfilled [28–30], partially fulfilled [29]. It was observed that all studies have demonstrated the results. Except in one study [28], all statistical methods were described (Fig 2A–2D).

With respect to financing and competing interests, in the source of funding criteria, two studies were considered as fulfilled [28, 29] and one as partially completed study [30]. None of the studies apparently showed any conflict of interest (Fig 2A–2D).

### Updates

Throughout the systematic review, some amendments were required to be made. We have, thus, included this topic concerning PRISMA 2020. One of these amendments was the update of the systematic review, given that data from one year had passed from the data to the first search (February 2020); Search strategy that follows in this search is the date of the first search and the update together in the flow diagram; No data was extracted as one of the criteria for analysis of the outcome of anti-inflammatory action of the plant/extract; More information on data extraction from *in vitro* studies has been added; Two authors resolving the discrepancies when arising.

## Discussion

In view of the systematic organization and analysis of scientific evidence of the anti-inflammatory effects of *L*. *ferrea* or *Caesalpinia ferrea* on *in vivo and* in *vitro* studies, we have sought to answer that part of the *L*. *ferrea* plant, and which type of extract has the most evident anti-inflammatory effects in the experimental models of acute inflammation on *in vivo* and *in vitro* studies.

Although the electronic research has identified one systematic review entitled Natural Antimicrobials and Oral Microorganisms: A Systematic Review on Herbal Interventions for the Eradication of Multispecies Oral Biofilms [31], that provide antimicrobial data from various medicinal plants, including *Caesalpinia ferrea*, the anti-inflammatory activity data were not described in said study. The originality of this study is, therefore, ratified. This would be the main strength of this research.

Nine studies [19–26, 30] have obtained the plants in the Northeastern region in Brazil, and four [12, 27, 28, 29] have obtained them in the Northern region which corroborates the literature data, which have demonstrated the wide distribution of this plant throughout Brazil, occurring in Caatinga, Atlantic Forest, and Cerrado domains especially in this area [3] and Northern region (AM, AP, PA, RO, RR) [32].

In this context, the Amazon region stands out, with a great diversity of plant species, where about 5,000 of the 35,000 plant species have great economic potential, either by the production of waxes, essential oils or by other constituents considered useful not only to humans, but also to the environment, animals and plants [33]. Brazil is the country with the greatest biodiversity on the planet (around 15% to 20%), of which, as plants are subsidies in the manufacture of medicines [34]. Among these plants, *L*. *ferrea* stands out and is the focus of research in this systematic review.

We have analyzed the methodological design of the ten *in vivo* studies and data described from the *in vitro* studies; it has been observed that the most used extract was aqueous extract. This has been found by Agra; Freitas; Barbosa-Filho (2007) whose study aimed to conduct a survey of plants and their modes of use for therapeutic purposes in northeastern Brazil. It has been demonstrated that the *L*. *ferrea* stem bark was used by decoction method or as an admixture solution [35].

In addition, the use of fruits left "soaking" and used for the treatment of influenza and bronchitis [36] has also been demonstrated. The study by Santos; Vilanova (2017) and Vásquez; Vásquez; de Mendonça; Noda (2014) has also demonstrated the use of leaf and fruit in the form of infusion and *in natura;* and the use of leaf and fruit in the preparation of tea, syrup, and macerated for the treatment of sore inflammation, sore throat, respectively [37, 38]. Infusion of leaves and fruits has also been demonstrated in the treatment of tuberculosis and liver inflammations in the Amazon region [1].

Regarding the anti-inflammatory effect, all the studies included in this systematic review have observed the existence of the anti-inflammatory activity of the plant, possibly independently of the part and/or type/fraction of the extract used. This is probably related to the fact that medicinal plants present some compounds (e.g., phenolic compounds) enabling anti-inflammatory action among various biological activities [39]. The presence of these and other compounds can be verified in fruits where gallic acid [4, 25], methyl gallate [4] and fatty acids [27, 28], have already been identified. For example, gallic acid regulates pro-inflammatory pathways, as the signaling pathway of nuclear factor kappa B (NF-κB) [40].

In addition, in the process of acute inflammation, inflammatory mediators are released. Mediators as cytokines and inflammatory proteins would act as biomarkers or predictors in the diagnosis and inflammatory diseases, respectively [41]. This has been observed in the modulation of TNF-α, IL-1β, NO and TGF-β controlling the inflammatory phase and also attenuating hypernociception in the wound healing study [20]. Anti-inflammatory activity could also occur via negative modulation, e.g., in carrageenan-induced paw edema, using the following mediators: bradykinin, nitric oxide, histamine, serotonin, and PGE_2_ [24].

This diversity in the several uses of the *L*. *ferrea* (extract and parts of the plant) as well the use of a great diversity of experimental models of inflammation, genus, species, animal number, and the number of animals by groups may cause difficulty in grouping the results by the similarity that makes impossible to demonstrate the sizes of the effect.

Exception by Pereira et al. (2016) who induced wounds on the animals and then administrated dressing contain the plant extract; all other *in vivo* studies have performed the treatment before inducing inflammation with the flogistic agent challenged to verify the anti-inflammatory action [20]. This conduct in the experimental designs differs from that applied in humans since the treatment is administrated after the onset of the disease. This is described as one of the challenges of the successful translations from animal models to the clinical environment in humans [42].

The principal limitations observed in the studies, object of this this systematic review (in accordance with the “unclear” risk of bias) were related to the risks of bias having to do with the concealment of the allocation, in addition to blinding of both the animals (induction of inflammation) and those, which they referred. The results have failed to indicate the groups to which they referred. Data on whether the animals had been properly randomized or not, and which method had been used were not provided in articles. Both this information and the execution of the blind assessment and the allocation concealment have helped reduce the impact of the bias on the experiments. These have enabled a reduction in the threats to the internal validity of the studies [43].

Limitations of this research are those inherent to systematic reviews of animal studies, such as the difficulty in the extraction of data, which are often presented in different ways in studies, especially when analyzing designs with high or unclear risk of bias. The authors of this research may have insufficiently interpreted the results presented in the included studies; the difficulty in collecting some data have not been taken into account, not all journals rely on some information, such as funding. Thus, in addition to the limitations inherent to preclinical studies, we still have these other limitations.

*In vitro* studies have been identified [28–30] ratifying the use of this type of experimental design to try to explain the mechanism of the action of anti-inflammatory drugs [44]. These studies could be translated into biomedical research when analyzed in more complex organisms [45]. However, it may be difficult to reflect the same results in terms of *in vivo* pharmacodynamics and pharmacokinetics studies [44].

Furthermore, quality analysis in preclinical studies without metanalysis is more challenging due to the subjectivity of the analyses. In addition, reporting the quality of *in vitro* studies followed the same principle of subjectivity in the analysis of the studies.

## Conclusions

Jucá (*L*. *ferrea*) appears to demonstrate anti-inflammatory activity regardless of the part of the plant and type of extract used in the experimental models and presents itself as a promising species in non-clinical research, thus corroborating its use in folk medicine for the treatment of inflammations. Although the evidence is considered as moderate by GRADEpro, a careful analysis of the results is important, given the presence of methodological bias. And the certainty of evidence is still insufficient to recommend the use of this plant in research.

For this reason, it is suggested preclinical studies in models of inflammation with greater methodological rigor based on standardized tools be designed for a more detailed evaluation of the effects of this plant of traditional use.

## Supporting information

S1 Table Prisma check list. (DOCX)

S2 Table Prisma abstract check list. (DOCX)

S1 Appendix Search strategy. (DOCX)

S2 Appendix Certainty of evidence in *in vivo*. (XLSX)
