# Supplementary material for: Libidibia ferrea (jucá) anti-inflammatory action: A systematic review of in vivo and in vitro studies
Source: PLoS One. 2021 Nov 5;16(11):e0259545. doi: 10.1371/journal.pone.0259545 (PMC8570521; doi:10.1371/journal.pone.0259545)
Supplement: S1 Appendix — (DOCX) [file pone.0259545.s003.docx]

**S1 Appendix: Search strategy** carried out in the databases on February 03, 2020

*Adjusted update on March 12, 2021: ("libidibia ferrea" OR "caesalpinia ferrea") AND ("antiinflammatory activity" OR "anti-inflammatory property" OR "anti-inflammatory effect" OR "antiinflammatory action").

| **Database** | **Search strategy** |
| --- | --- |
| **PUBMED** | ("Libidibia ferrea"[All Fields] OR Libidibia[All Fields] OR "Caesalpinia ferrea"[All Fields] OR ("caesalpinia"[MeSH Terms] OR "caesalpinia"[All Fields]) OR juca[All Fields] OR "pau-ferro"[All Fields]) AND ("anti inflammatory activity"[All Fields] OR "anti-inflammatory activities"[All Fields] OR "anti-inflammatory property"[All Fields] OR "anti inflammatory properties"[All Fields] OR "anti-inflammatory effect"[All Fields] OR "anti-inflammatory effects"[All Fields] OR "anti-inflammatory action"[All Fields] OR "anti-inflammatory actions"[All Fields]) |
| **SCIENCE DIRECT** | ("libidibia ferrea" OR libidibia OR "caesalpinia ferrea" OR caesalpinia OR juca OR "pau-ferro") AND ("anti-inflammatory activity" OR "anti inflammatory property" OR "anti-inflammatory effect" OR "anti inflammatory action")***** |
| **WEB OF SCIENCE** | ("libidibia ferrea" OR libidibia OR "caesalpinia ferrea" OR caesalpinia OR juca OR "pau-ferro") AND ("anti-inflammatory activity" OR "anti inflammatory activities" OR "anti-inflammatory property" OR "anti inflammatory properties" OR "anti-inflammatory effect" OR "anti inflammatory effects" OR "anti-inflammatory action" OR "anti inflammatory actions") |
| **LILACS** | 1^st^ search: ("libidibia ferrea" OR libidibia OR "caesalpinia ferrea" OR caesalpinia OR juca OR "pau-ferro") AND ("anti-inflammatory activity" OR "atividade anti-inflamatória" OR "actividad antiinflamatoria") 2^nd^ search: ("libidibia ferrea" OR libidibia OR "caesalpinia ferrea" OR caesalpinia OR juca OR "pau-ferro") AND ("anti-inflammatory property" OR "propriedade anti-inflamatória" OR "propriedad antiinflamatoria") 3^th^ search: ("libidibia ferrea" OR libidibia OR "caesalpinia ferrea" OR caesalpinia OR juca OR "pau-ferro") AND ("anti-inflammatory effect" OR "efeito anti-inflamatório" OR "efecto antiinflamatorio") 4^th^ search: ("libidibia ferrea" OR libidibia OR "caesalpinia ferrea" OR caesalpinia OR juca OR "pau-ferro") AND ("anti-inflammatory action" OR "ação anti-inflamatória" OR "acción antiinflamatoria") |
| **SCOPUS** | ("libidibia ferrea" OR libidibia OR "caesalpinia ferrea" OR caesalpinia OR juca OR "pau-ferro") AND ("anti-inflammatory activity" OR "anti inflammatory property" OR "anti-inflammatory effect" OR "anti inflammatory action") |
| **GOOGLE SCHOLAR** | ("Libidibia ferrea" OR "Caesalpinia ferrea" OR juca OR "pau-ferro") AND ("anti-inflammatory activity" OR "anti-inflammatory property" OR "anti inflammatory effect" OR "anti-inflammatory action") |
| **PROQUEST** | (libidibia NEAR/2 ferrea OR caesalpinia NEAR/2 ferrea OR juca OR pau ferro) AND (anti-inflammatory NEAR/2 activity OR anti-inflammatory NEAR/2 property OR anti-inflammatory NEAR/2 effect OR anti inflammatory NEAR/2 action) |
